# Transcriptomic Profiling of *Arabidopsis thaliana* Mutant *pad*2.1 in Response to Combined Cold and Osmotic Stress

**DOI:** 10.1371/journal.pone.0122690

**Published:** 2015-03-30

**Authors:** Deepak Kumar, Riddhi Datta, Saptarshi Hazra, Asma Sultana, Ria Mukhopadhyay, Sharmila Chattopadhyay

**Affiliations:** Plant Biology Laboratory, Drug Development/Diagnostics & Biotechnology Division, CSIR- Indian Institute of Chemical Biology, 4, Raja S.C. Mullick Road, Kolkata, 700032, India; Chinese Academy of Sciences, CHINA

## Abstract

The contribution of glutathione (GSH) in stress tolerance, defense response and antioxidant signaling is an established fact. In this study transcriptome analysis of *pad*2.1, an *Arabidopsis thaliana* mutant, after combined osmotic and cold stress treatment has been performed to explore the intricate position of GSH in the stress and defense signaling network *in planta*. Microarray data revealed the differential regulation of about 1674 genes in *pad*2.1 amongst which 973 and 701 were significantly up- and down-regulated respectively. Gene enrichment, functional pathway analysis by DAVID and MapMan analysis identified various stress and defense related genes viz. members of heat shock protein family, *peptidyl prolyl isomerase (PPIase)*, *thioredoxin peroxidase* (*TPX*2), *glutathione-S-transferase* (*GST*), *NBS-LRR* type resistance protein etc. as down-regulated. The expression pattern of the above mentioned stress and defense related genes and *APETALA* were also validated by comparative proteomic analysis of combined stress treated Col-0 and *pad*2.1. Functional annotation noted down-regulation of *UDP-glycosyl transferase*, *4-coumarate CoA ligase* 8, *cinnamyl alcohol dehydrogenase* 4 *(CAD*4*)*, *ACC synthase* and *ACC oxidase* which are the important enzymes of phenylpropanoid, lignin and ethylene (ET) biosynthetic pathway respectively. Since the only difference between Col-0 (Wild type) and *pad*2.1 is the content of GSH, so, this study suggested that in addition to its association with specific stress responsive genes and proteins, GSH provides tolerance to plants by its involvement with phenylpropanoid, lignin and ET biosynthesis under stress conditions.

## Introduction

Plants are consistently exposed to unfavourable growth conditions throughout their life cycle. To ensure survival, plants must effectively and efficiently sense, respond, and adapt to their ever-changing environment. As we all know that the environmental stresses can have a devastating effect on plant metabolism, disrupting cellular homeostasis and uncoupling major physiological processes. Till date, the molecular mechanisms associated with signal transduction, leading to changes in gene expression in environmental stress response, are largely unknown.

Unique structural properties, abundance redox potential and wide distribution in most living organisms have been drawn significant attention towards the role of GSH [1,2]. In plant tissues reduced form of glutathione i.e. GSH which is also considered as redox buffer is mainly present at 2 to 3 mM concentrations compared to its oxidized form, glutathione disulfide (GSSG) (10–200 μM) [3–5]. Together, it can be noted that GSH, a tripeptide thiol, is an important metabolite with a broad spectrum of functions, and its homeostasis is essential to maintain cellular redox potential and effective responses to stress in plants [6].

The role of GSH in plant defense has long been established [7,8]. Enhanced resistance to *Fusarium oxysporum* due to increase in GSH level has also been revealed in melon and tomato roots [9]. Ball et al., (2004) [3] reported that 32 stress-responsive genes were altered due to changed GSH metabolism in *Arabidopsis rax*1-1 and *cad*2-1. *Arabidopsis* mutants of γ-*ECS*, viz. *pad*2.1, with only 22% of wild-type amounts of GSH, were found susceptible to *Pseudomonas syringae* as well as *Phytophthora brassicae* [10,11]. Furthermore it was reported that the GSH deficiency in *pad*2.1 affects defense-related signaling events, which confers a susceptibility to pathogens [12]. It has also been reported that GSH acts as a signaling molecule and mitigates biotic stress through NPR1-dependent/independent salicylic acid (SA)-mediated pathway [13–15]. The relevance of the GSH levels in protecting maize against chilling-induced injury has already been established [16]. Previously it was reported that the enzymes of GSH biosynthesis and metabolism were induced together in response to stress [17]. The role of GSH in the detoxification of heavy metals has also been investigated [18].

GSH contents modulation transmits information through diverse signaling mechanisms, including the establishment of an appropriate redox potential for thiol/disulphide exchange and the release of calcium to the cytosol [19]. Earlier reports have also indicated the role of GSH in protecting cells against stress effects through the activation of various defense mechanisms due to its involvement in redox signaling [20–22]. This indicates that there is significant overlap in the signal transduction cascades that induce GSH synthesis and those involved in defense functions that use GSH, such as GST and glutathione peroxidase (GPX), some of which show a particularly strong response to ROS [23]. In defense related signaling pathway, GSH supposed to interact with ROS, redox molecules like thioredoxins (Trxs) and glutaredoxins (Grxs) and plant hormones (SA, ABA). The redox dependence of the pathway suggests that any biotic or abiotic stimulus that can perturb the cellular redox state could up-regulate the same set of defense genes via the NPR1 mediated pathway [24,25]. One of our recent investigation also depicted that GSH was supposed to act through multistep signaling pathways to mitigate environmental stresses [26]. Collectively it can be said that GSH has a significant role in environmental stress tolerance, in combination with other established signaling molecules.

In addition to the contribution of GSH in defense, its contribution to other essential functions has been reported as well. For example, effect of GSH content in the growth of roots of *Arabidopsis* was also reported, a recent study reported the contribution of GSH to wheat somatic embryo development signifying that GSH is essential for somatic embryogenesis [27,28].

In this study, we investigated *pad*2.1, a GSH depleted mutant, transcriptome and proteome in response to combined stress treatment with a view to identify the genes and proteins altered under changed GSH conditions to combat stresses. Additionally, the identification of differentially expressed genes from this study provides essential information on probable candidates involved with GSH to mitigate environmental stresses in plant and offers clues for future study to unravel the intricate position of GSH in plant defense.

## Materials and Methods

### *Arabidopsis* seed germination and combined stress treatment

The *Arabidopsis* seeds procured from NASC [Nottingham *Arabidopsis* Stock Centre—Col-0 (N-1092) and *pad*2.1 (N-3804)] were surface sterilized and grown in Murashige and Skoog (MS) [29] medium and maintained in growth chamber at 22°C under 16 h light/8 h dark cycles. For combined osmotic and cold stress treatment, 3 weeks old seedlings were further placed in filter paper for 5 min at 4°C for early dehydration with cold stress [30] and then transferred to semisolid MSO medium with 30% PEG at 4°C for an additional 6 hours; the leaves were collected for RNA and protein isolation. Morphological changes after stress treatment in Col-0 and *pad*2.1 were also monitored.

### Estimation of GSH and GSH:GSSG ratio

GSH was extracted from leaves of control and stress treated Col-0 and *pad*2.1 as well quantified [31]. GSH:GSSG ratio was measured according to Ishikawa et al. (2010)[4]. The experiments were repeated with six biological replicates.

### RNA extraction and microarray analysis

Total RNA was isolated from leaf tissue of plants for each experiment. Each experiment consisted of combined stress treated Col-0 and *pad*2.1 samples in two replicates. For microarray experiment total RNA was extracted and purified from 300 mg of leaf material using the RNeasy Plant Minikit (Qiagen’s RNeasy Minikit) and eluted in RNAse-free water. For semi-quantitative and quantitative RT-PCR RNA was isolated from leaf samples ground in liquid nitrogen and finally extracted by TriZol method. RNA integrity was analyzed via formaldehyde agarose gel electrophoresis. RNA quality and quantity were checked with a NanoDrop ND-1000 spectrophotometer and an Agilent 2100 Bioanalyzer. Samples were prepared according to the protocols outlined in the GeneChip Expression Analysis Technical Manual and hybridizations to the Agilent custom *Arabidopsis* 8x60k microarray designed by Genotypic technology private limited (AMADID: 48015), Bangalore, India. Taken as a whole the gene expression of the combined stress treated Col-0 and *pad*2.1 was compared. Microarray Data analysis and normalization have been done using GeneSpring GX version 12.0 and Microsoft Excel. The transcriptomics data were deposited at GEO (Gene Expression Omnibus) at the National Centre for Biotechnology Information 1 (NCBI, http://www.ncbi.nlm.nih.gov/geo/) with GSE61170 accession number.

### Expression profiling by semi-quantitative and quantitative RT-PCR analysis

The effect of combined stress treatment on Col-0 and *pad*2.1 for the expression of stress and defense related genes were recorded by semi-quantitative RT-PCR. Total RNAs from both control and combined stress treated Col-0 and *pad*2.1, leaf tissue were isolated using TriZol reagent (Invitrogen) and about 1 μg of total RNA of each sample was reverse-transcribed by RevertAid first strand cDNA synthesis kit (Fermentas) using oligo (dT) as primer. The PCR was carried out using the following thermal cycling profile: 95°C for 3 min, followed by required number of cycles (95°C for 30 sec, 58°C for 30 sec, and 72°C for 45 sec). The sequences of the primer pairs listed in Table 1. The PCR products and their sizes were examined using 1% agarose gel electrophoresis. *Actin* gene was amplified as an endogenous loading control for testing the validity of template preparation. Semi-quantitative RT-PCR products were quantified according to relative abundance of the band by QuantityOne software (BIORAD). The expression of each gene was confirmed in at least three rounds of independent RT-PCR reactions. The quantitative RT-PCR was performed using Roche LightCycler 96 System (Roche Applied Science, USA) with FastStart Essential DNA Green Master (Roche, USA). Quantitative RT-PCR was performed for selected genes presented in Table 1. Amplification was performed for 40 cycles at 94°C, 30 sec and 60°C, 2:30 min with a preceding initial denaturation of 30 sec at 95°C. The constitutively expressed *actin* gene was used as reference gene.

**Table 1:** List of primers used in the quantitative and semi-quantitative RT-PCR.

Genes	Forward Primer (5’- 3’)	Reverse Primer (5’-3’)	Amplicon size in bp (Ref. No.)
*ACO1*	GATCAAAGAGAGAGAGATGGAGA	TGAAATGTTTGGGATCTGACAGAT	326 (NM_127517.4)
ACS*7*	TGTTTGAAAGGGAACGCAGG	TTCGTCGGTCCATGAACTCA	244 (AF332390.1)
*Actin*	GGCTGATGGTGAAGATATTCAAC	CATTGTAGAAAGTATGATGCCAGA	281 (NM_115235.3)
*APETALA*2	ATGTGGGATCTAAACGACGCAC	ACCCGCGGACGATCCGGGGCT	306 (NM_001204009.1)
*APX*1	ATGACGAAGAACTACCCAACC	TCAGGATAAGTACCCAAGCTC	700 (NM_001084012)
*CAD*4	ATGGGAAGTGTAGAAGCAGGA	TACCGCAGCATCCAACGACC	307 (NM_112832.3)
*GST*	ATGGCAGGAATCAAAGTTTTC	CGACTCAATTTCAATGCCCATGG	311 (NM_100174.2)
*NBS-LRR*	ATGTTCAGATCGAACGCAAGA	CTAGATGACTTGTTGACTGAAA	302 (NM_118856.1)
*TPX*2	ATGGCTCCAATTACTGTCGGC	AAACTTCACATGCTTGTTCTCTG	300 (NM_105269.4)
*HSP*70	ATGTTGGGAATGAGAACTGTGT	TCTCACCCATATACCGAAGCCG	306 (NM_101038.4)
*Osmotin*	ATGGCAAACCTCTTGGTCTCT	GATCGGGAGCTGGTGGGAT	711 (NM_117234.2)
*HSP*18.2	ATGTCTCTCATTCCAAGCATT	TCAATTAGCCCCGGAGATAT	486 (NM_125364.2)
*ADC*2	ATGCCTGCTTTAGCTTGCGTT	TCATCTTCTGGCTCAGCTCAA	712 (NM_119637.2)
*ERF*5	ATGGCGACTCCTAACGAAGTA	CGTCTCTCTTCCGTTTCTTCT	700 (NM_124094.2)
*ERD*	ATGGCTACAATAAACGATATTGGA	GATCCTCAAGTAGACAACAGAAT	303 (NM_117632.4)
*MYB*59	ATGACTCCACAAGAAGAGCG	CTCTCTCCATATGTCATCCA	300 (NM_180894.3)
*CYP*79B2	ATGAACACTTTTACCTCAAA	GTTTCCTAACTTCACGCATG	300 (NM_120158.2)

### Functional enrichment and annotation

Functional enrichment of differentially expressed genes was analyzed by Database for Annotation, Visualization and Integrated Discovery (DAVID) v6 [32] and singular enrichment analysis (SEA) with the agriGO tool [33]. A cut off *P* value of 0.05 was used for enriched pathways and gene ontology functions by DAVID. GO term enrichment was computed by SEA analysis in the selected set of genes by comparing it to the reference set (in this case, the Agilent 3 Oligo Microarray (GPL2871). The statistical method used is the Fisher test. The Benjamini-Yekutieli method is used to do the multiple comparison correction.

### Assignment of the differentially regulated genes to functional pathways by MapMan and DAVID

Genes identified by the microarray analysis were analyzed to identify relevant functional pathways by MapMan software (http://gabi.rzpd.de/projects/MapMan, version 3.5.1) [34] and (DAVID) v6.

### Protein isolation and 2-DE

Total protein was isolated from 1.5 g of leaf tissue using phenol extraction method. In brief, the tissue was ground in liquid nitrogen and suspended in extraction buffer (700 mM Sucrose, 500 mM Tris–HCl, pH7.5, 50 mM EDTA, 100 mM KCl, 2% (w/v) β-mercaptoethanol, 1 mM phenylmethylsulfonyl fluoride) and protein extraction was done following standard protocol. The isolated protein was resuspended in IEF buffer consisting of 7 M urea, 2 M thiourea, 4% 3-[(3-cholamido propyl)-dimethylammonio]- 1-propane sulfonate (CHAPS), 20 mM DTT and 1% (w/v) Bio-Lyte (3/10) ampholyte (BioRad Laboratories, Hercules,CA, USA) as standardized before [35,36]. The protein concentration was determined by Bradford’s method [37]. 100 μg of protein was used to passively re-hydrate immobilized pH gradient strip (7 cm; pH4–7; BioRad Laboratories, Hercules, CA, USA) for 12 h. IEF was performed as follows: 250 V for 30 min, 4000 V for 2 h, 4000 V for 10000 V-h, 500 V for 1 h on BioRad PROTEAN IEF Cell system (BioRad Laboratories, Hercules, CA, USA). Focused strips were then equilibrated in equilibration buffers I & II (BioRad Laboratories, Hercules, CA, USA) for 15 min each. For running gels in the second dimension, 12% SDS polyacrylamide gels were used and stained with colloidal Coomasie Brilliant Blue (CBB) G-250 [38].

### Image analysis

Using Versa-Doc Image system the gel images were taken (BioRad Laboratories, Hercules, CA, USA) and the images were analyzed with PD Quest software version 8.0.1 (BioRad Laboratories, Hercules, CA, USA). Spot detection was performed by matching the gels automatically and manual verification. Densities of spots were normalized against whole gel densities. The spots detected in at least two replicate gels were selected for annotation; the percentage volume of each spot was averaged for the different (three biological replicates of combined stress treated Col-0 and *pad*2.1) Using Versa-Doc Image system the gel images were taken (BioRad Laboratories, Hercules, CA, USA) and the images were analyzed with PD Quest software version 8.0.1 (BioRad Laboratories, Hercules, CA, USA). Spot detection was performed by matching the gels automatically and manual verification. Densities of spots were normalized against whole gel densities. The spots detected in at least two replicate gels were selected for annotation; the percentage volume of each spot was averaged for the different (three biological replicates of combined stress treated Col-0 and *pad*2.1) gels and significant protein fold changes between the samples were statistically evaluated using the *t*-test function implemented in the software. Spots showing differences between control and treated leaves with a *P* value of <0.05 were chosen for further analysis.

### In-gel digestion and mass spectrometric analysis

Manually excised spots from 2D gels were digested with trypsin (in-gel trypsin digestion kit, Pierce, USA) following the manufacturer's protocol. Using Zip-Tip μ-C18 (Millipore, Billerica, MA) the samples were desalted, and analyzed using 4800 MALDI TOF/TOF analyzer (Applied Biosystems, Foster City, CA, USA). The 0.5 μl dissolved sample in a solvent consisting of 0.1% trifluoroacetate and 50% acetonitrile (ACN) in MilliQ was mixed with 0.5 μl of matrix solution (1mg/ml α-cyano-4-hydroxycinnamic acid dissolved in the aforementioned solvent), applied to a 384-MALDI sample target plate, and dried in air. After that by using a delayed extraction approach the peptides were evaporated with a ND:YAG laser at 355 nm. The peptides were accelerated with 25 kV injection pulse for time of flight analysis. Each spectrum was the cumulative average of 1000 laser shots. The MS/MS spectrum was collected in MS/MS 1 kV positive reflectron mode with fragments generated by post source decay (PSD). The mass tolerance of MS/MS was set to ±20 ppm. Following processing, 10 MS/MS precursors were selected (Minimum signal to noise ratio-50). The instrument was calibrated with the Applied Biosystems 4700 Proteomics Analyzer Calibration Mixture before each analysis. By using GPS Explorer Software (Applied Biosystems) data interpretation was done, and an automated database search was performed by using the MASCOT program (Matrix Science Ltd., London, U.K).

### Statistical analysis

The experiment were repeated at least three times and the data presented as the mean±standard error (SE) to compare the GSH content, GSH:GSSG ratio and relative gene expression profile of Col-0 and *pad*2.1 under control and stress conditions. Statistical analysis was performed using one-way analysis of variance (ANOVA) followed by Student-Newman-Keuls multiple comparison test (GraphPad InStat software, ver. 3.1). A *P* value corresponding to 0.05 or 0.01 or 0.001 was considered to be statistically significant.

## Results

### Morphological analysis and Confirmation of stress response at transcript level

Morphological changes were noted in leaves of *pad*2.1 after stress treatment and were compared with Col-0. Results showed that leaves were more wilted in comparison to that of Col-0 (S1A Fig.). Further, to evaluate the molecular response, semi-quantitative RT-PCR analysis were performed and results were noted with the reduced expression of stress responsive genes like *osmotin*, *HSP*70, *arginine decarboxylase* 2 (*ADC*2) and *ascorbate peroxidase* 1 (*APX*1) by 0.5, 0.73, 0.77 and 0.78 fold respectively in combined stress treated *pad*2.1. Higher expressions of *HSP*18.2, *ERF*5 and *APETALA*2 by 2.0, 2.0 and 4.33 fold respectively, were noted in combined stress treated *pad*2.1 compared to that of Col-0 seedlings (S1B Fig.).

### Effect of stress on GSH content and GSH:GSSG ratio

**Fig 1:**
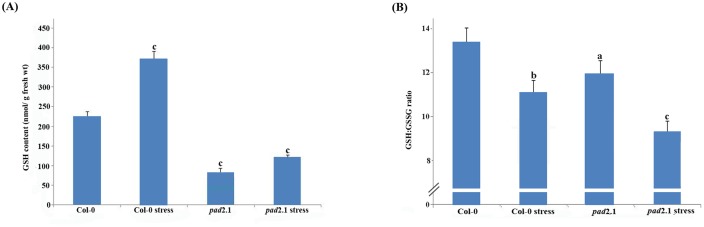
Effect of stress treatment on (A) GSH content and (B) GSH:GSSG ratio in Col-0 and *pad*2.1. Data are presented as mean ± SE (n = 3). Lower case letters indicate significant difference from Col-0 at ^*a*^ *P*<0.05, ^*b*^ *P*<0.01 and ^*c*^ *P*<0.001 (Student-Newman-Keuls multiple comparison test).

After stress treatment 64.8% increase in GSH content was observed in stress treated Col-0 in comparison to control condition. In *pad*2.1 only 41.6% increase in GSH content was noted after stress treatment in comparison to control. GSH:GSSG ratio also found higher in stress treated Col-0 than *pad*2.1 (Fig. 1).

### Microarray experiment

**Fig 2:**
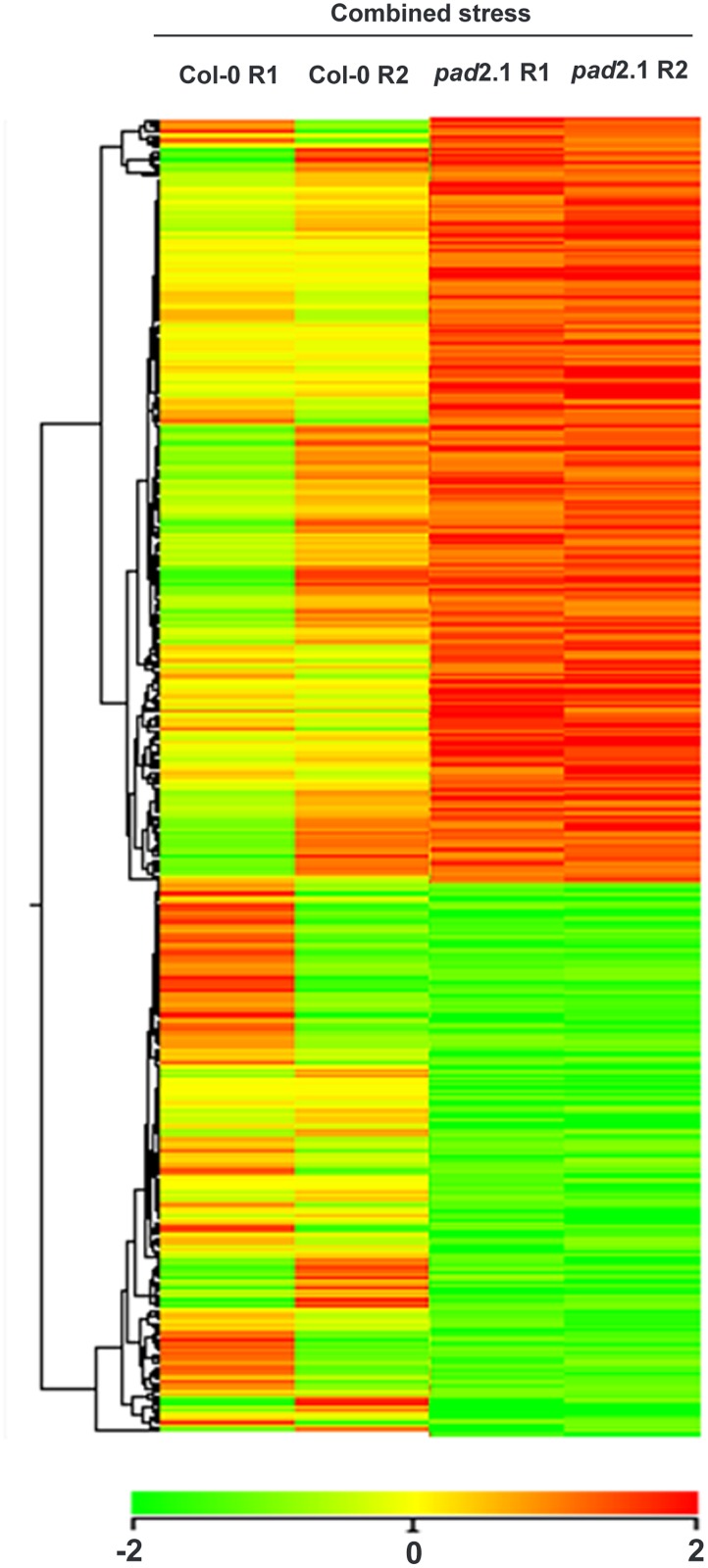
Heat map with hierarchical cluster tree for differentially expressed genes. Tree classified on the basis of gene expression. Over-expressed (log2 fold change > = 1) and under-expressed (log2 fold change < = -1) genes are shown by red and green colour respectively.

To investigate the transcript changes in response to combined stress treatment in *pad*2.1, the microarray analysis of the combined stress treated *pad*2.1 seedling was performed as compared to combined stress treated Col-0. From the results, it was evident that the transcript level responses of *pad*2.1 to this treatment were massive. Images were quantified using Agilent Feature Extraction Software and the extracted raw data was analyzed using Agilent GeneSpring GX software. Normalization of the data was done in GeneSpring GX using the 75th percentile shift (Percentile shift normalization is a global normalization, where the locations of all the spot intensities in an array are adjusted). This normalization takes each column in an experiment independently, and computes the percentile of the expression values for this array, across all spots (where n has a range from 0–100 and n = 75 is the median). It subtracts this value from the expression value of each entity) and normalized to specific control samples. About 1674 genes were found significantly differentially expressed in *pad*2.1 among which 973 significantly up-regulated (log2 fold change > = 1) and 701 significantly down-regulated (log2 fold change < = -1) genes were identified (S1 Table) and hierarchical heat map image has been generated which showed genes expression profile of combined stress treated *pad*2.1 compared to combined stress treated Col-0 (Fig. 2). Statistical significance of the differential gene expression was measured by Student’s *t*-test and *P* values were adjusted for false discovery rate correction using Benjamini Hochberg method. Genes were classified based on functional category and pathways using DAVID biological interpretation tool (http://david.abcc.ncifcrf.gov/). The quality control results of the experiment in the form of PCA (principal component analysis) have been shown in S2 Fig. PCA on conditions is mainly used to visually assess the quality of replicates in a biological sample. Each point in the 3D plot represents a sample. The scores are used to check data quality (blue: combined stress treated *pad*2.1, green: combined stress treated Col-0).

### Analysis of differentially regulated genes to functional pathways by DAVID

973 up- and 701 down-regulated genes were considered for analysis using DAVID. As listed in S2 Table, the down-regulated genes were associated with 30 metabolic and biosynthetic pathways viz. stilbenoid, diarylheptanoid and gingerol biosynthesis, terpenoid, glutathione, amino sugar and nucleotide sugar metabolism, biosynthesis of phenylpropanoid, phenylalanine, amino acyl-tRNA and fatty acid biosynthesis, cystein, methionine, aspartate, glutamate, glycine, serine and threonine metabolism.

**Fig 3:**
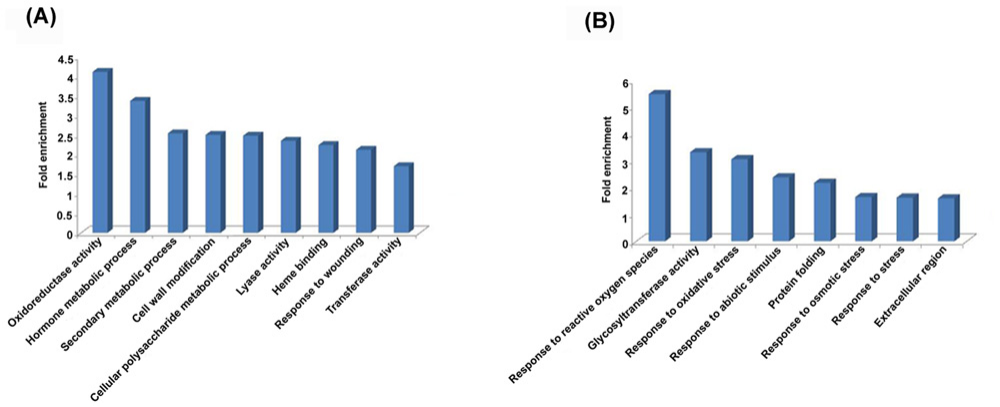
DAVID functional annotation categories of differentially expressed genes. Significantly enriched categories for (A) up-regulated genes and (B) down-regulated genes in *pad*2.1.

**Fig 4:**
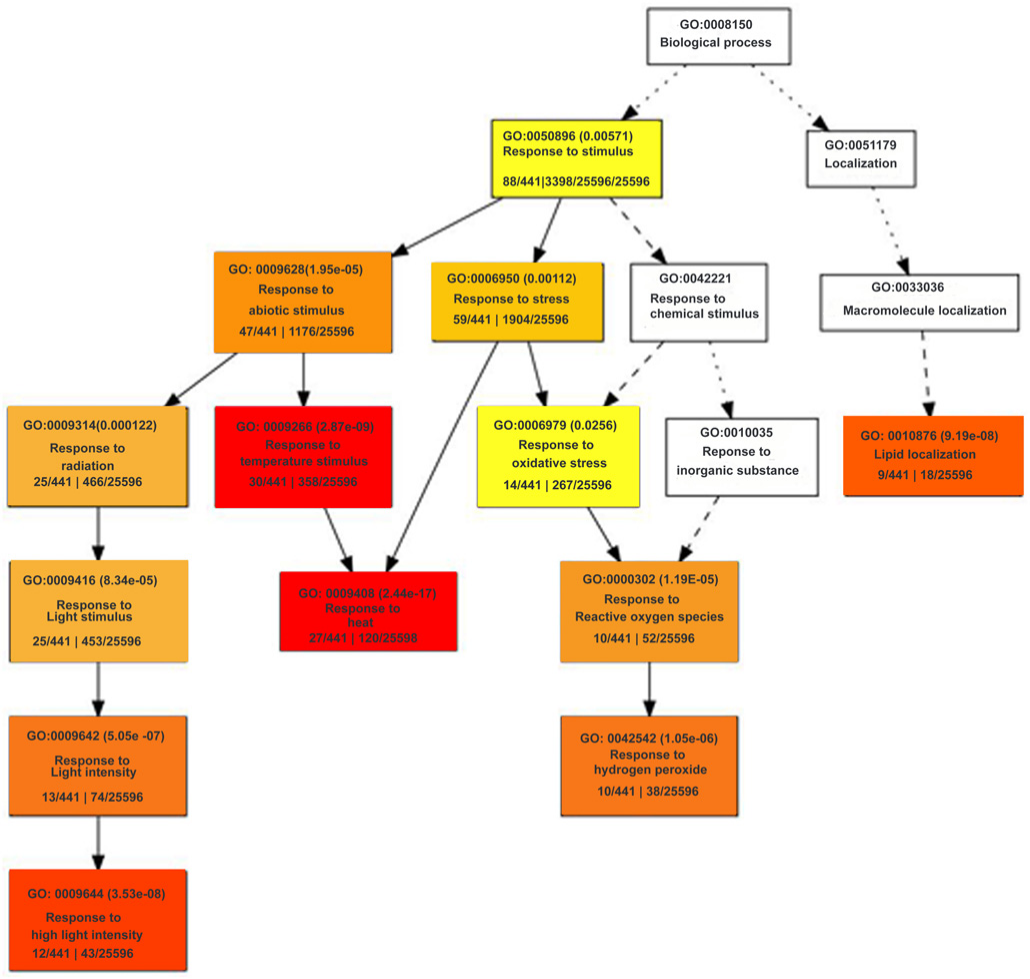
Hierarchical tree graph of over-represented GO terms in down-regulated genes by singular enrichment analysis generated by agriGO. Boxes in the graph show GO terms labelled by their GO ID, term definition and statistical information. The significant terms (adjusted *P*<0.05) are marked with color, while non-significant terms are shown as white boxes. The degree of color saturation of a box correlates positively with the enrichment level of the term. Solid, dashed and dotted lines represent two, one and zero enriched terms at both ends connected by the line, respectively. The rank direction of the graph runs from top to bottom.

DAVID was also used for gene ontology analysis and term enrichment for various biological processes, molecular function and cellular component as shown in S3 Table. Among DAVID functional annotation categories of differentially expressed genes, significantly enriched categories for up-regulated genes in *pad*2.1 were oxidoreductase activity, hormone metabolic process, secondary metabolic process, cell wall modification, polysaccharide metabolic process, lyase activity, heme binding, response to wounding, transferase activity and significantly enriched categories for down-regulated genes were response to reactive oxygen species, glycosyl transferase activity, response to oxidative stress, response to abiotic stimulus, protein folding, response to osmotic stress, response to stress stimulus and have been shown in Fig. 3. As observed by singular enrichment analysis by agriGo (Fig. 4) the most significantly reduced biological process was response to stress stimulus like reactive oxygen species, heat, high light intensity and radiation in *pad*2.1.

**Fig 5:**
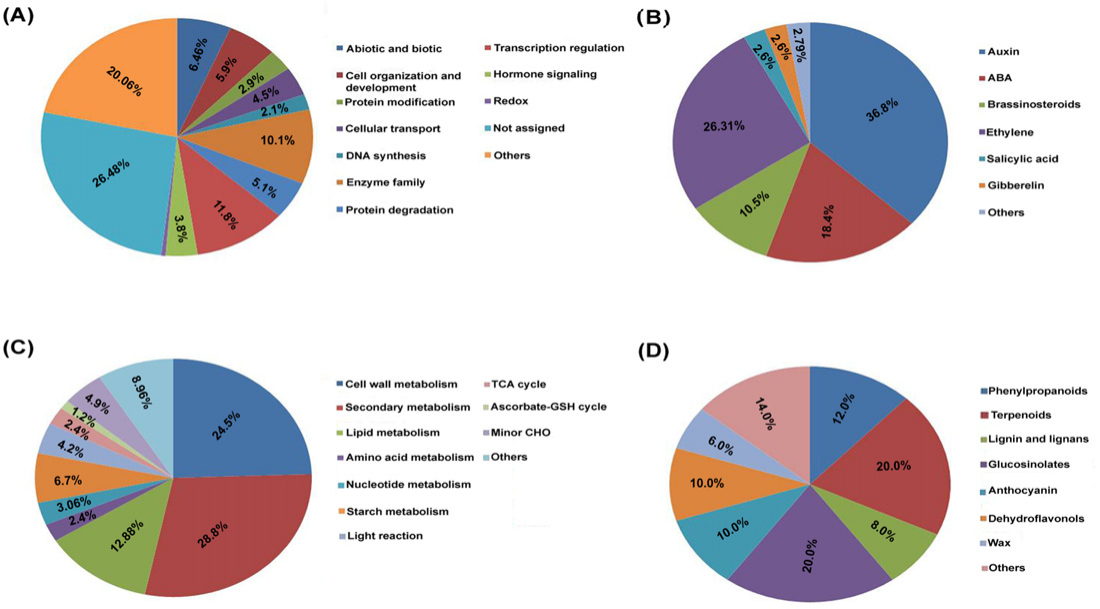
In response to combined stress treatment, (A) Functional annotation of the differentially expressed gene in Col-0 and *pad*2.1 through MapMan. Differentially expressed genes related to (B) hormones, (C) metabolic pathways and (D) secondary metabolites in Col-0 and *pad*2.1.

Functional annotation of the identified genes through MapMan revealed that majority of them were related to regulation of transcription, enzyme families, abiotic and biotic stress stimulus, cell organization and protein degradation categories (Fig. 5A). The genes related to hormones were further classified, among which most of them were related to auxin, ET, ABA and brassinosteroid (Fig. 5B). Metabolism mapping also revealed that among differentially expressed genes largely of them were related to cell wall metabolism, secondary metabolism, lipid metabolism and starch metabolism (Fig. 5C). The secondary metabolism related genes were further classified which showed majority of them were related to phenylpropanoids, terpenoids, lignin and lignans, glucosinolates, anthocyanin and dehydroflavonols (Fig. 5D). The stress response as well as metabolism overview visualization by MapMan analysis in response to GSH feeding reveals substantial alteration in the transcriptome.

**Fig 6:**
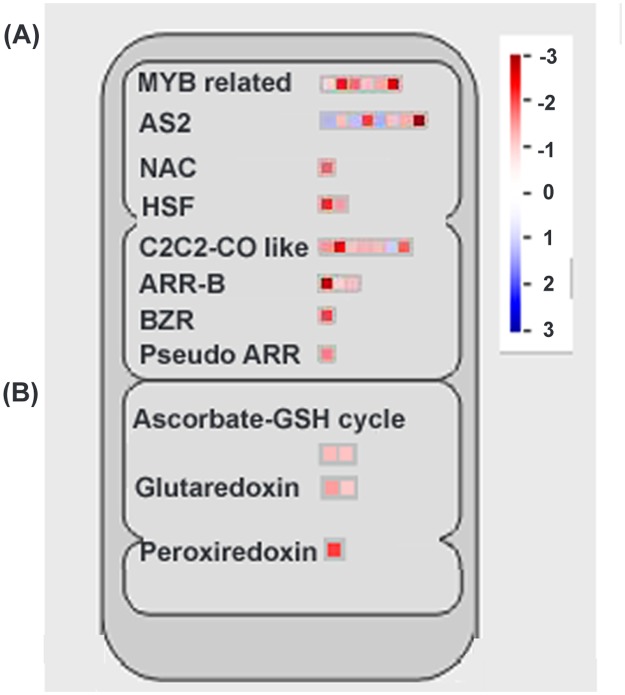
MapMan analysis of different (A) transcription factors (B) cellular redox maintaining genes. Up-regulated genes in blue and down-regulated genes in red colour respectively.

According to MapMan analysis of differentially expressed genes, various transcription factors were found differentially regulated in response to combined stress treatment under altered GSH condition (Fig. 6A). Among which *NAC*, *HSF* (Heat shock transcription family), *MYB*-related, *MADS* (MADS box transcription factor family), *ARR-B*, *C2C2-CO-like* (B-box zinc finger family), *AS*2 (LOB-domain containing protein), *BZR*1 (brassinazole-resistant 1 protein) and Pseudo-ARR transcriptional factor proteins were found down-regulated in *pad*2.1 under combined stress condition.

**Fig 7:**
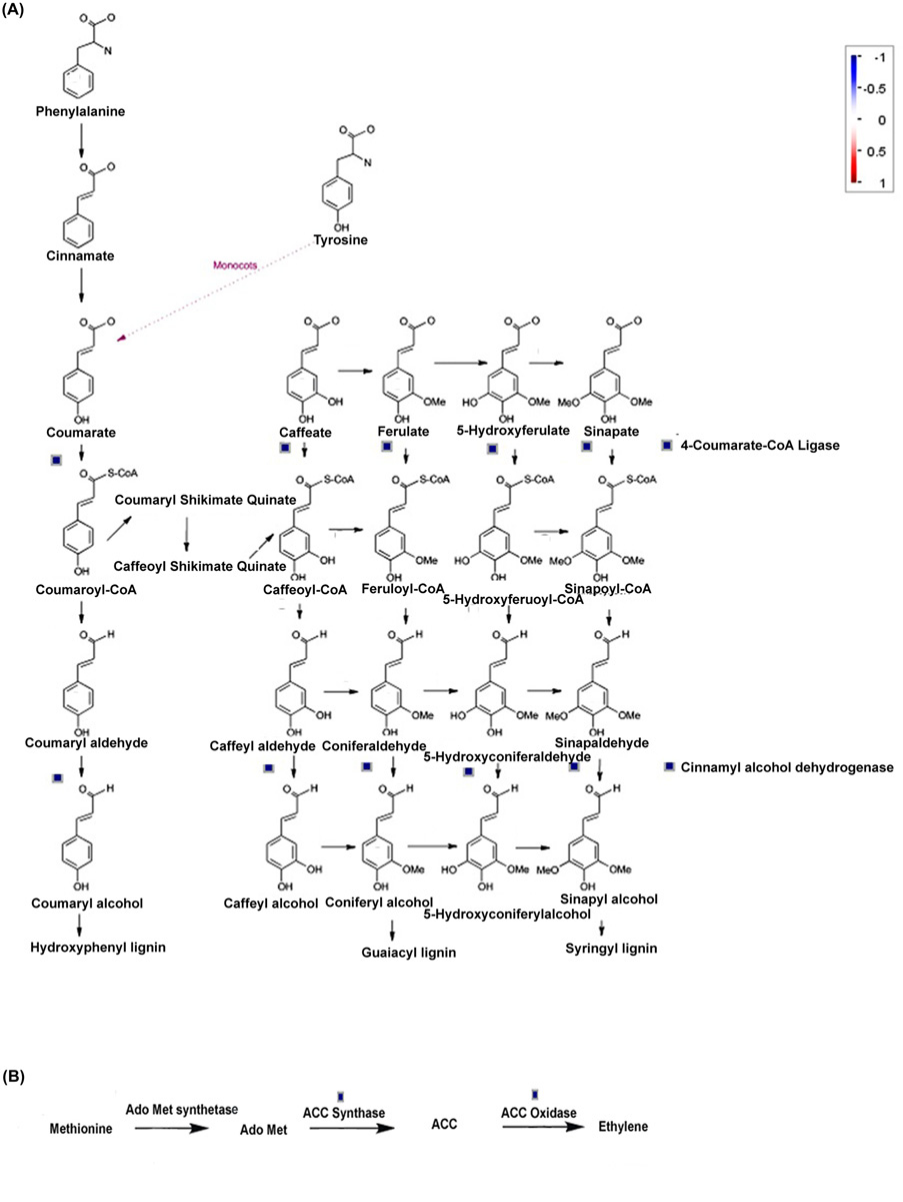
Identified genes related to (A) phenylpropanoid and lignin (B) ET biosynthetic pathways by MapMan analysis. Up-regulated genes in red and down-regulated genes in blue colour respectively.

MapMan analysis of differentially expressed genes also revealed that phenylpropanoid, lignin, ET, anthocyanin and dihydroflavonols biosynthetic pathways were affected in *pad*2.1 following combined stress stimulus. Genes involved in phenylpropanoid and lignin biosysnthesis like *UDP-glycosyl transferase*, *4-coumarate CoA ligase* 8 and *CAD*4 were found down-regulated in *pad*2.1 after combined stress treatment (Fig. 7A). Genes of ET biosynthetic pathway like *ACC oxidase* and *ACC synthase* were found down-accumulated in combined stress treated *pad*2.1 (Fig. 7B). MapMan analysis of differentially expresssed genes also revealed the up-regulation of anthocyanin and dihydroflavonols biosynthetic pathway related genes in *pad*2.1.

### Comparative proteomics analysis

**Fig 8:**
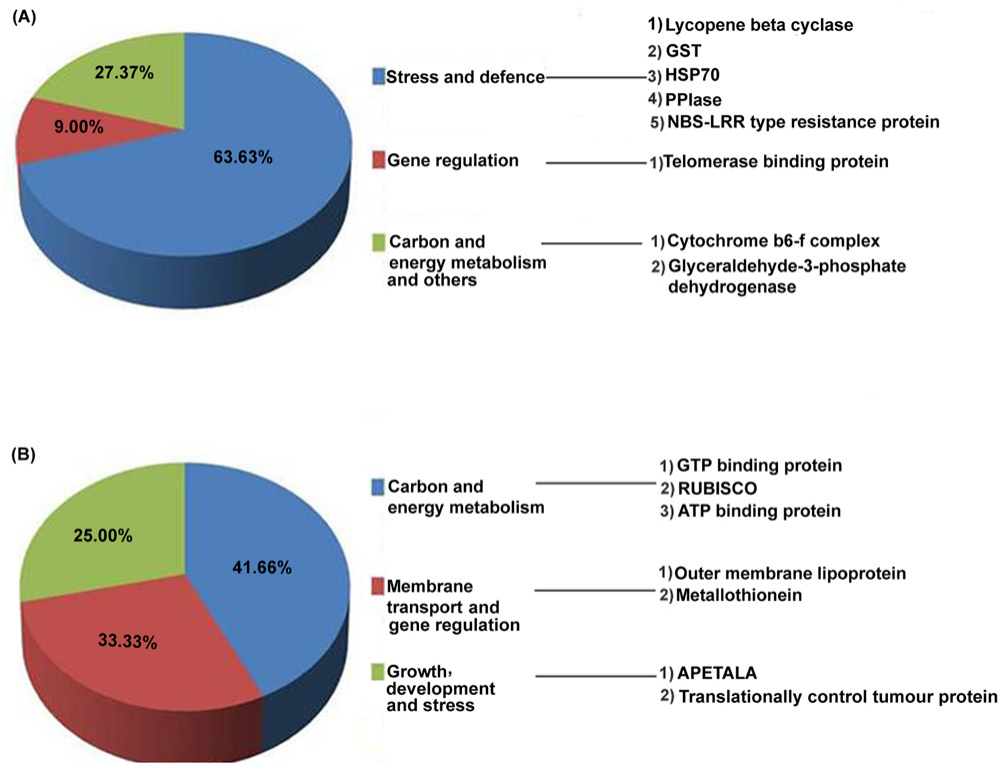
Functionally categorized (A) down-accumulated and (B) up-accumulated proteins in response to combined stress treatment in *pad*2.1.

**Fig 9:**
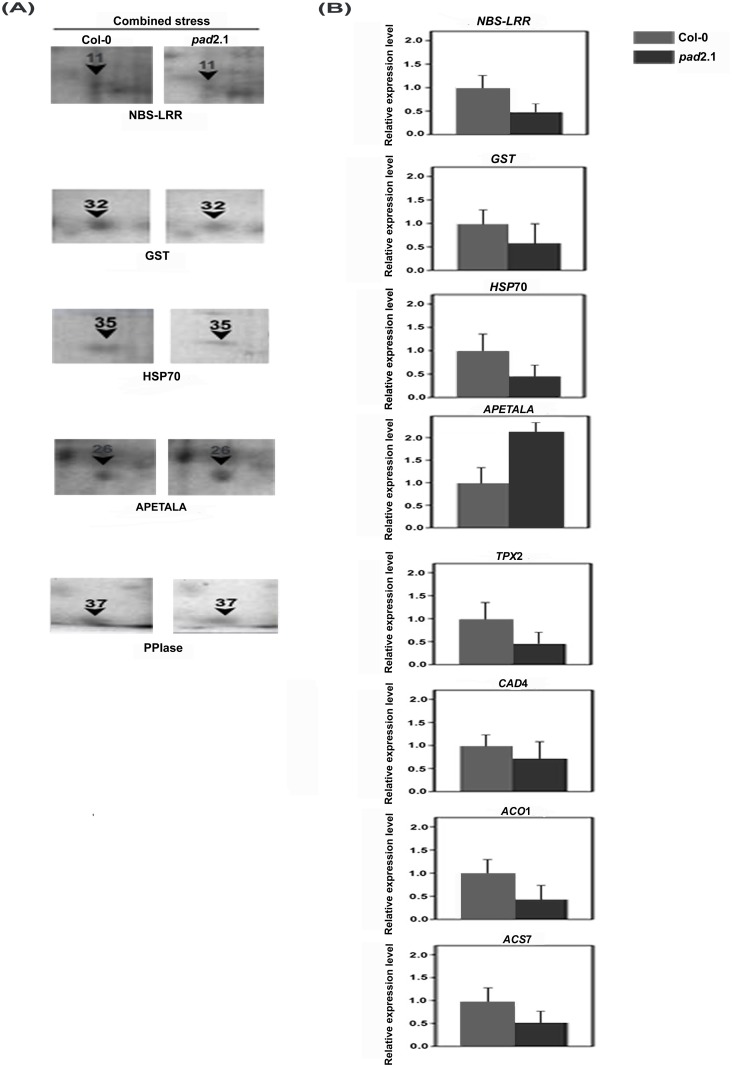
Validation of some differentially expressed genes of microarray experiments by (A) comparative proteomic analysis (B) Quantitative RT-PCR. Data are presented as mean ± SE (n = 3). *P*<0.05 (Student’s *t*-test).

Comparative proteomics was conducted from the protein isolated from combined stress treated Col-0 and *pad*2.1. The differentially accumulated spots as identified by comparative protein profiling (S3 Fig.) were further identified. Approximately 289 and 278 spots were identified in combined stress treated Col-0 and *pad*2.1 respectively. Out of which 155 spots were similar with the overall coefficient of variation being 33.72 between combined stress treated Col-0 and *pad*2.1, respectively. Out of 23 differentially expressed and identified proteins (S4 Table), 11 were found down-accumulated in response to combined stress in *pad*2.1 and about 63.63% of them viz. HSP70, NBS-LRR type resistance protein, lycopene cyclase, GST, PPIase etc. were related to stress and defense, whereas, 27.37% were related to both carbon and energy metabolism with other proteins (Fig. 8A). Rest 12 proteins were found up-accumulated in *pad*2.1 and amongst which most of them were related to carbon and energy metabolism (Fig. 8B). Down-accumulation of some of the above mentioned proteins also validated the differential expression of similar genes in response to combined stress treatment in *pad*2.1 (Fig. 9A-B, Table 2).

**Table 2:** MALDI TOF-TOF MSMS based identification of proteins which validated differential expression of five genes in *pad*2.1 in response to combined stress.

SSP no.^a^ (Spot no.)	Th. Mr/pI Exp. Mr/pI ^b^	Avg Fold change^c^	Protein (Taxonomy)	Accession No. ^d^	Mascot Score ^e^	Sequence Coverage (%) ^f^
3803 (11)	96.0/6.60 61.80/5.70	0.49	NBS-LRR type resistance protein (*Triticum monococum*)	gi|33321037	37	7
7205 (32)	24.10/5.92 27.60/6.60	0.71	Glutathione-S-transferase (*Arabidopsis thaliana)*	gi|13194824	145	54
1904 (35)	76.99/5.17 79.00/5.20	0.10	Heat shock protein-70 (*A*. *thaliana*)	gi|24030296	358	25
8003 (37)	28.20/8.80 14.70/6.80	0.03	Peptidyl-prolyl isomerase (*A*. *thaliana*)	gi|6899901	108	34
6402 (26)	49.30/6.28 36.20/6.30	2.15	Sterile APETALA (*A*. *thaliana*)	gi|9758652	50	10

Identified differentially accumulated proteins in Col-0 and *pad*2.1 plants in response to combined stress treatment.

^a^Assigned sample protein and spot number as indicated in Fig. 8A and S3 Fig.

^b^Th Mr/pI, theoretical mass of protein in kDa and pI; Exp Mr/pI, experimental mass of protein in kDa and pI.

^c^Average spot intensity fold change.

^d^NCBI accession number of identified protein spots.

^e^Statistical probability of true positive identification of predicted proteins calculated by MASCOT (http://www.matrixscience.com).

^f^Sequence coverage%: percentage of predicted protein sequence covered by matched peptides.

### Validation of some differentially expressed genes by quantitative RT-PCR

Differentially expressed genes like *NBS-LRR*, *HSP*70, *APETALA*, *TPX*2, *GST*, *CAD*4, *ACO*1 and *ACS*7 were validated by quantitative RT-PCR. All the above mentioned genes except *APETALA* were found down-regulated by almost 2 fold expression change in stress treated *pad*2.1 in comparison to that of in Col-0 (Fig. 9B). Genes like *GST*, *NBS-LRR*, *TPX*2 and *ACO*1 were found down-regulated whereas *APETALA* and *CYP79*B2 were noted up-regulated in *pad*2.1 under control condition. Rest of them like *ACS*7, *ERD* and *CAD*4 did not show much change in their expression in Col-0 and *pad*2.1 under control condition (S4 Fig.).

## Discussion

In response to stress condition in *pad*2.1 there is a possibility of more ROS production. *HSPs* and other stress responsive genes were found up-regulated in response to stress and it was also reported earlier that these genes were induced by oxidative stress in Col-0. In case of *pad*2.1 after combined stress treatment there can be two possibilities which may affect the differential expression of genes. First, a possible reason for differential expression of genes may be an increase in ROS or oxidative stress in plant cells due to GSH depletion. Second, high GSH content can have a direct or indirect role in induction or regulation of the gene and protein expressions by thiol mediated modulations of various transcription factors, subunits of RNA polymerase and protein kinase [39] which were previously reported to be induced by ROS or oxidative stress, and have been found down-regulated in *pad*2.1.

### Effect on transcription factors

The expression of NAC transcription factor family gene was induced by osmotic, drought, high salinity, and abscisic acid. Microarray analysis of transgenic plants overexpressing NAC family transcription factors revealed that several stress-inducible genes were up-regulated in the transgenic plants, and the plants showed significantly increased drought tolerance [40,41].

Glutathione depletion inhibited the heat and the reagent initiated activation of the heat shock factor 1 (HSF1) and did not promote the expression of *HSP*70 mRNA [42]. It was also demonstrated that *A*. *thaliana* plants with increased *HSFA1b* expression showed increased productivity and harvest index under water deplete and water-limiting conditions [43]. Myb related transcription factors were also found up-regulated in *A*. *thaliana* in response to abiotic stress [44]. Role of various MYB family proteins under biotic and abiotic stress conditions elucidated in previous reports can be corroborated with our data [45–47].

During water-stress, PSARK::IPT plants displayed increased expression of brassinosteroid-related genes like *BZR*1, *BAK*1 and *BRI*1 which were responsible for its tolerance to drought stress [48]. MADS-box transcription factors, besides being involved in floral organ specification, have also been implicated in several aspects of plant growth and development. Expression levels of four MADS box genes were up-regulated by more than two folds in response to cold and dehydration stress treatments [49].

*AS*2 is an important auxin responsive factor was found highly induced under salinity and drought stress treatment in sorghum [50]. *Arabidopsis* mutant *cad*2 showed less auxin biosynthesis and transport due to depleted glutathione which might affect the expression of *AS*2 [51].

Above mentioned transcription factors were found up-regulated under stress condition but in *pad*2.1 they are found down-regulated in response to combined stress treatment. It indicated that GSH may be directly or indirectly induces the expression of these transcription factors by thiol mediated modification of their inducers. But due to less content of GSH in *pad*2.1, there were the possibility of lesser thiol mediated modification and their activation.

### Effect on stress response

In the present investigation genes like *PP2-*A3 (phloem protein 2-LIKE A3), *PDF*1.4 (defensin-like protein 19), at5g48595, at5g23035, *MLO-like protein* 9 and *NBS-LRR* type disease resistant proteins were found down-regulated in combined stress treated *pad*2.1. The main characteristic of these PR-family members is its antimicrobial activity against a large number of phytopathogenic species, including fungi and bacteria [52]. MLO-like protein 9 involved in perception of bacterial and fungal infection like *Pseudomonas* and powdery mildew, likewise the *NBS-LRR* genes are involved in pathogen recognition and defense signaling in *Arabidopsis* [53]. At protein level, disease resistance protein like NBS-LRR protein (spot no. 11) was also found down-accumulated in combined stress treated *pad*2.1. The above mentioned down-regulated genes and protein are supposed to be directly or indirectly induced by GSH.

Among the differentially expressed genes in response to combined stress treatment in *pad*2.1, *NDR1/HIN1-1ike* genes and *toll-interleukin 1 receptor* (TIR) were found up-regulated in *pad*2.1. The role of *TIR* in combating biotic stress in *Arabidopsis* has already been established [54]. It was reported earlier that TIR domain plays a crucial role in a cell death signaling pathway [55,56]. Excessive oxidative stress in response to pathogenesis has been reported to be associated with toll-like receptor (TLR) activation in humun neutrophil tissues [57].

**Fig 10:**
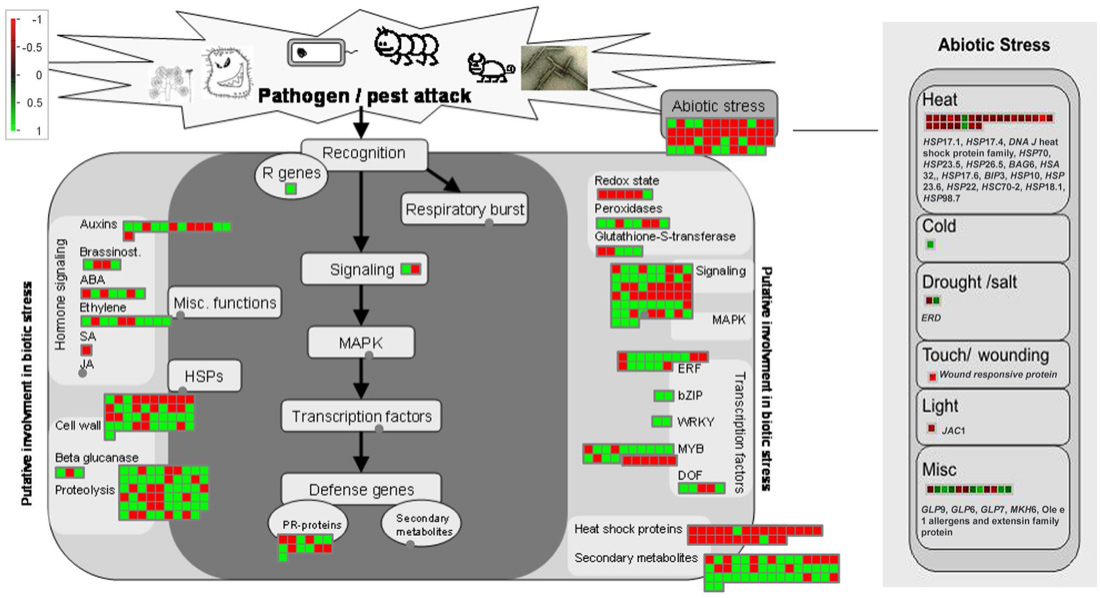
Stress response overview of transcriptome altered in response to combined stress treatment in *pad*2.1 as visualized by MapMan analysis.

Abiotic stress responsive genes like members of heat shock protein family, *ERD* (early responsive to dehydration stress), at4g10270, *JAC (*J-domain protein required for chloroplast accumulation response 1) and *PPIase* were found down-regulated in *pad*2.1 in response to combined stress treatment (Fig. 10). All cytoplasmic and mitochondrial members of HSP70 family were strongly induced by low temperature [58]. Correlation between GSH oxidation and *HSP*s induction under stress condition has been already established [59]. Previously, *ERD*11 and *ERD*13 were isolated from a cDNA library from *A*. *thaliana* plants dehydrated for 1 h were sequenced and characterized [60]. Earlier *ERD*9 was found repressed in response to low GSH and ascorbate [61]. Compared to the drought-susceptible cultivar, the osmotic stress-induced expression of total *PPIase* activity in different tissues of sorghum was found dramatically higher in the cultivar which was tolerant to drought [62]. The water stress induced enhancement in the *PPIase* levels in different tissues of the Col-0 may be helping the other stress-induced proteins to maturation by virtue of their chaperonic and PPIase activity. It is also likely that enhanced levels of PPIase activity under stress conditions may be regulating the expression of other genes imparting stress tolerance since they are also implicated in signal transduction. In validation of transcriptomics data, HSP70 and PPIase (spot no. 35, 37) proteins were also found down-accumulated in *pad*2.1 (Fig. 8A) in response to combined stress treatment. It has been already reported that *pad*2.1 contains only 22% of GSH compared to Col-0. So, it can be assumed that genes and proteins which were found down-regulated in response to stress condition are due to the altered level of GSH in *pad*2.1 as compared to Col-0.

In previous investigations, after stress treatment development of redox imbalance in plant cell has been reported. Excessively produced GSH develops more reduced state in plant cell which might lead to the modulation of proteins as noted in NPR1 where the reduction of disulfide bond results the NPR1 monomers which then moved to the nucleus and consequently induced the stress and defense related genes [25]. The high concentration of GSH is supposed to regulate the gene expression by thiol mediated modulation of transcription factors, RNA polymerase subunits, and protein kinases [39].

### Effect on redox related genes and proteins

Among the redox related genes *GST*, *glutaredoxin*, *monothiol glutaredoxin*, *TPX*2 and *APX*2 were found down-regulated in response to combined stress treatment in *pad*2.1 (Fig. 6B). GST catalyzes the conjugation of GSH with hydrophobic toxic compounds to form derivatives that can be secreted from the cell, sequestered in the vacuole, or catabolized [63]. Apart from conjugation reaction, GST isoenzymes perform other GSH-dependent catalytic activities which include the reduction of organic hydroperoxides [64]. Glutaredoxins generally catalyzes reduction reaction by using the GSH/glutathione reductase system in photosynthetic organisms [65,66]. Peroxidase activity of glutaredoxins and its contribution to the resistance to oxidative stress has already been established [67–70]. H_2_O_2_ feeding through the transpiration stream induced *APX*2 expression in *A*. *thaliana* and protected the leaves against subsequent photoinhibition and foliar oxidative damage [71]. TPX was reported sensitive to hyperoxidation under oxidative stress and inactivation of TPX activity was important to allow thioredoxin to reduce other substrates under conditions of acute oxidative stress [72]. In *pad*2.1, due to low GSH content more production of ROS after combined stress treatment is expected and this excessive ROS production might down-regulated the expression of *TPX*2. In the case of other genes of this group ROS induced GSH is supposed to act as their direct or indirect inducer. Among these down-regulated genes of this group GST (spot no. 32) was also found down-accumulated at protein expression level in combined stress treated *pad*2.1.

### Effect on phenylpropanoid, lignin and ET pathways

Phenylpropanoid like sinapyl alcohol, coniferyl alcohol and coumaryl alcohol are the important precursors of lignin biosynthesis. The role of lignin in defense response in plants has been already established [73]. Previous study has shown that lignin biosynthesis was induced by GSH treatment in bean cell culture [74]. Hence, down-regulation of important lignin biosynthesis genes as identified in our results further confirmed the distinct role of GSH in lignin biosynthesis. ACS is the enzyme of rate limiting step in ET biosynthesis [75]. In our previous report we have already proposed that GSH has an important role in inducing ET biosynthesis [26]. Present result supports the previous report about the role of GSH in inducing ET biosynthesis.

### Effect on dihydroflavonols and anthocyanins pathways

Due to less GSH content in *pad*2.1, stress treatment might develop oxidative stress which was supposed to induce the expression of genes like *anthocyanin 5-aromatic acyltransferase*, *leucoanthocyanidin dioxygenase* (*LDOX*), *anthocyanidin synthase*. *dehydroflavonols 4-reductase* (*DFR*) and *flavonoid 3-O-glucosyltransferase*. The involvement of H_2_O_2_ on the accumulation of anthocyanin in rice seedlings has been reported earlier [76]. Since, anthocyanin has also been reported as an antioxidant [77], plants, under depleted GSH condition i.e. *pad*2.1, induced the genes of anthocyanin and dihydroflavonols pathways, to combat stress as noted here.

## Conclusions

Collectively, this study reports an inclusive transcriptomic and proteomic analysis of *A*. *thaliana* mutant *pad*2.1 in response to combined treatment of cold and osmotic stress. Down-regulation of selected stress-responsive genes in *pad*2.1 under stress conditions confirmed the dynamic contribution of GSH in plant stress and defense. Furthermore, down-regulation of ET-related genes point towards the role of GSH in ET biosynthesis under stress. Additionally this study opens a new insight into the interaction of GSH with other defense related secondary metabolic pathway, particularly phenylpropanoid, lignin, anthocyanin and dihydroflavonols biosynthetic pathway, which might be notable information in plant defense.

## Supporting Information

S1 Fig Effect of stress treatment on the (A) leaf morphology and (B) expression of stress and defense related genes in Col-0 and *pad*2.1. Data are presented as mean ± SE (n = 3). Lower case letters indicate significant difference from that of Col-0 at ^*a*^ *P*<0.05, ^*b*^ *P*<0.01 and ^*c*^ *P*<0.001, ns-not significant (Student-Newman-Keuls multiple comparison test).
(TIF)

S2 Fig Principal component analysis (PCA) of microarray experiment. The PCA components represented the X, Y and Z axes (green: combined stress treated Col-0, blue: combined stress treated *pad*2.1).
(TIF)

S3 Fig Representative 2-DE gel images of combined stress treated Col-0 and *pad*2.1. (TIF)

S4 Fig Study of the expression of various genes by quantitative RT-PCR in both control and stress treated Col-0 and *pad*2.1 which are found differentially expressed in microarray experiment. Data are presented as mean ± SE (n = 3). Lower case letters indicate significant difference from that of Col-0 at ^*a*^ *P*<0.05, ^*b*^ *P*<0.01 and ^*c*^ *P*<0.001, ns-not significant (Student-Newman-Keuls multiple comparison test).
(TIF)

S1 Table List of Identified (A) up-regulated (red) and (B) down-regulated genes (green) (*P*< 0.05) by microarray analysis in combined stress treated *pad*2.1. (XLSX)

S2 Table Identified (A) up-regulated and (B) down-regulated functional pathway related genes analysed by DAVID in combined stress treated *pad*2.1. (XLSX)

S3 Table Gene ontology annotation analysis of the (A) up-regulated and (B) down-regulated genes in combined stress treated *pad*2.1 (BP-Biological process, CC-Cellular component, MF-Molecular function). (XLSX)

S4 Table List of identified up-accumulated and down-accumulated proteins in response combined stress treatment in *pad*2.1. (XLSX)
