# Supplementary figures and images for: Transcriptomic Profiling of Arabidopsis thaliana Mutant pad2.1 in Response to Combined Cold and Osmotic Stress
Source: PLoS One. 2015 Mar 30;10(3):e0122690. doi: 10.1371/journal.pone.0122690 (PMC4379064; doi:10.1371/journal.pone.0122690)

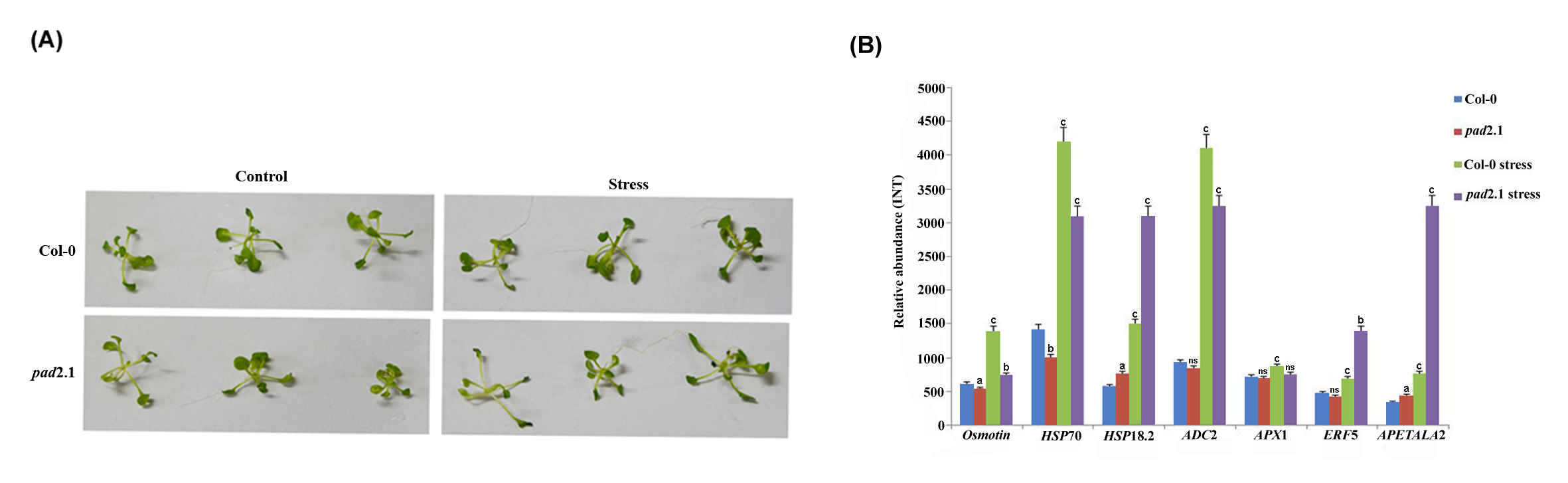

Supplement: S1 Fig — Data are presented as mean ± SE (n = 3). Lower case letters indicate significant difference from that of Col-0 at a P<0.05, b P<0.01 and c P<0.001, ns-not significant (Student-Newman-Keuls multiple comparison test). (TIF) [file pone.0122690.s001.tif]

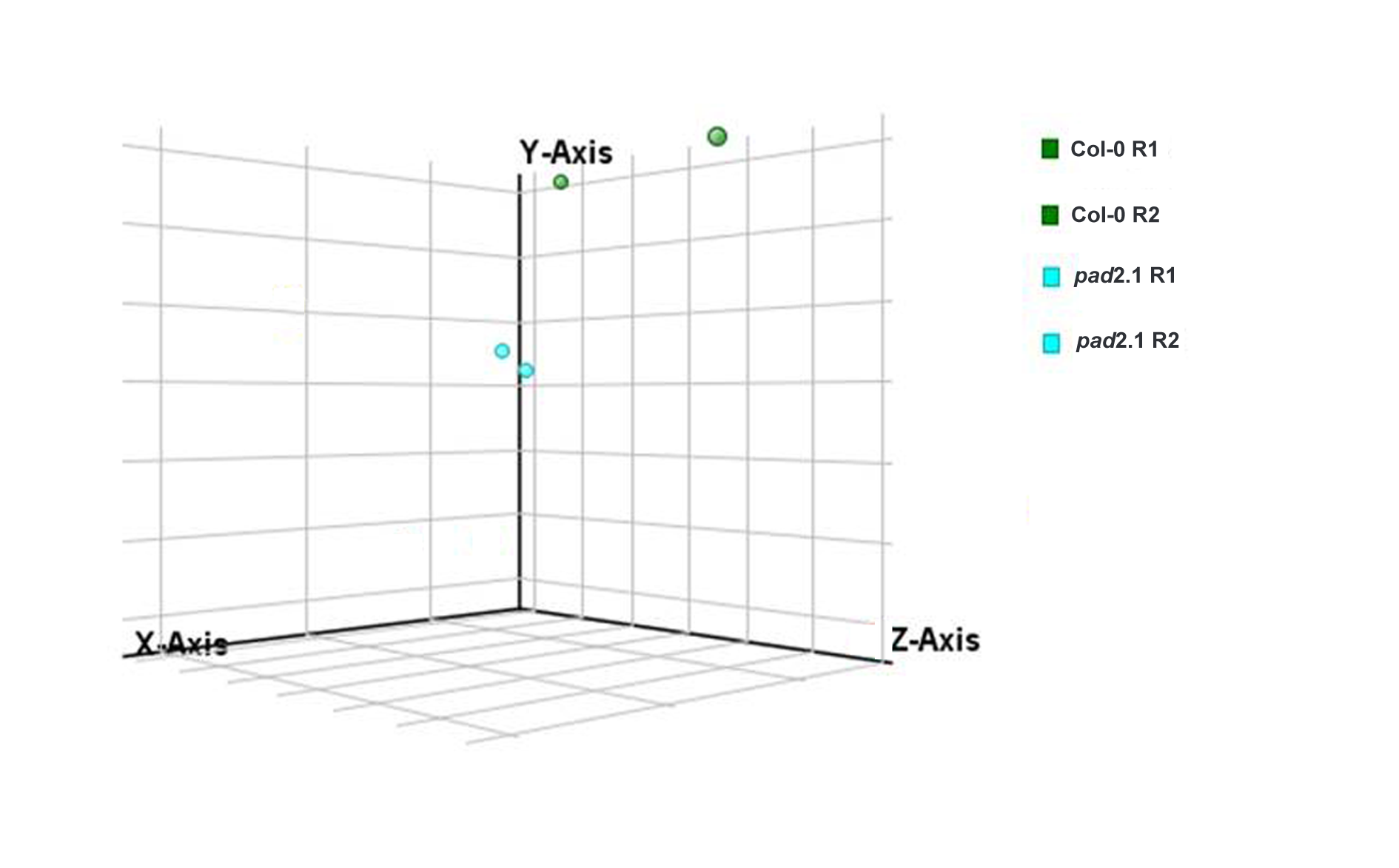

Supplement: S2 Fig — The PCA components represented the X, Y and Z axes (green: combined stress treated Col-0, blue: combined stress treated pad2.1). (TIF) [file pone.0122690.s002.tif]

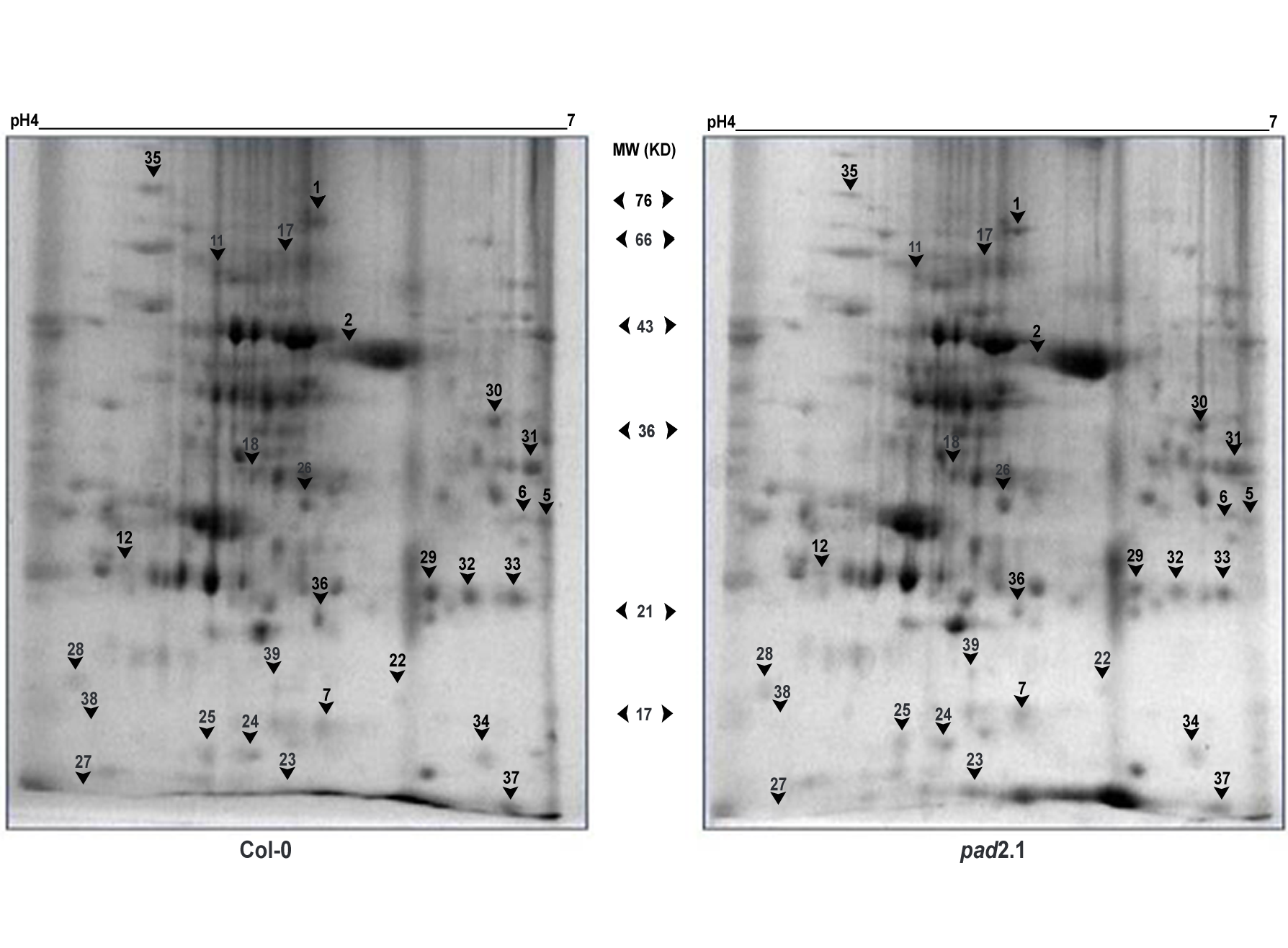

Supplement: S3 Fig — (TIF) [file pone.0122690.s003.tif]

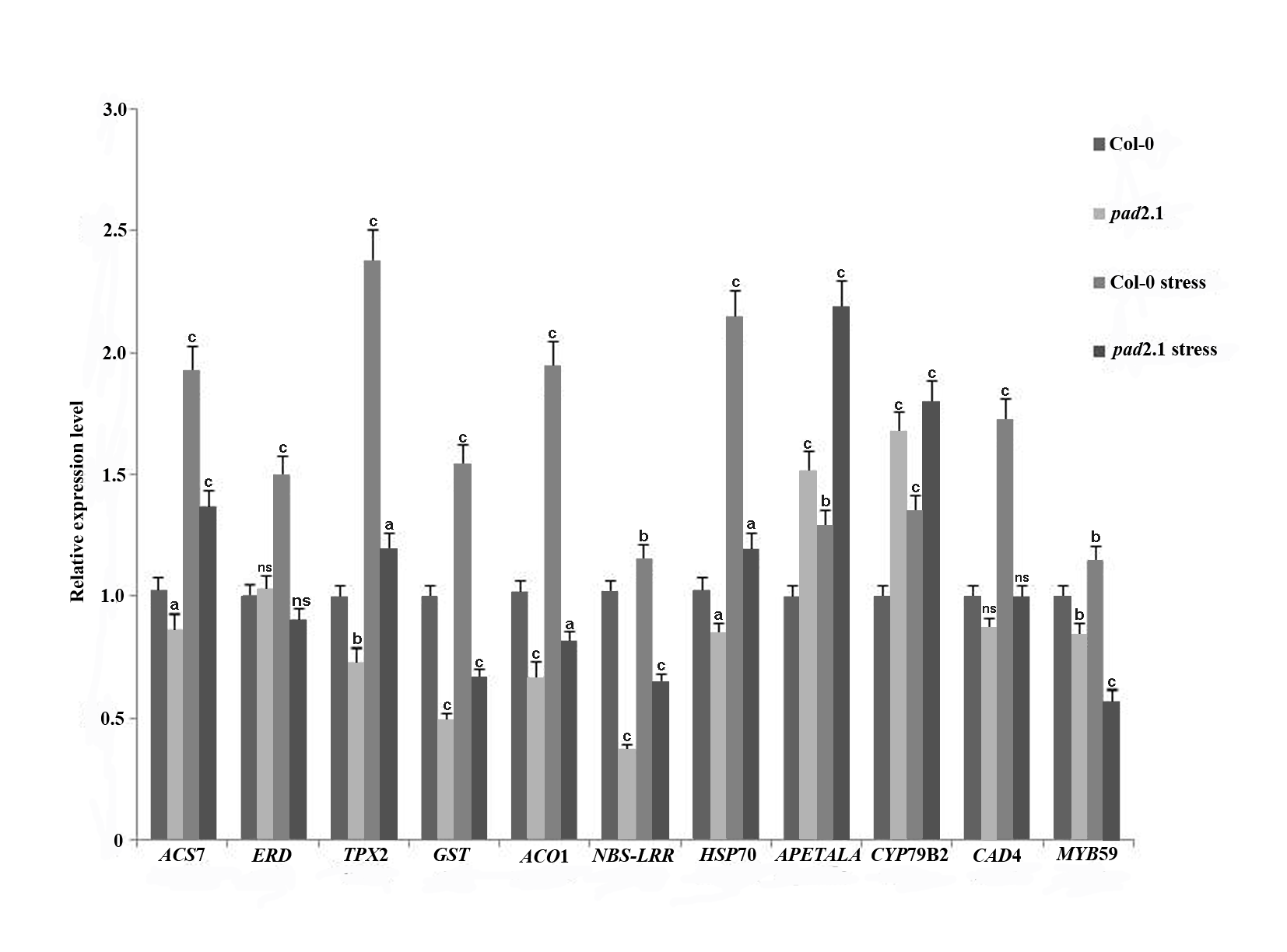

Supplement: S4 Fig — Data are presented as mean ± SE (n = 3). Lower case letters indicate significant difference from that of Col-0 at a P<0.05, b P<0.01 and c P<0.001, ns-not significant (Student-Newman-Keuls multiple comparison test). (TIF) [file pone.0122690.s004.tif]
